# Facilitating the Recruitment of Minority Ethnic People into Research: Qualitative Case Study of South Asians and Asthma

**DOI:** 10.1371/journal.pmed.1000148

**Published:** 2009-10-13

**Authors:** Aziz Sheikh, Laila Halani, Raj Bhopal, Gopalakrishnan Netuveli, Martyn R. Partridge, Josip Car, Chris Griffiths, Mark Levy

**Affiliations:** 1 Allergy & Respiratory Research Group, Centre for Population Health Sciences, University of Edinburgh, Edinburgh, United Kingdom; 2 Centre for Population Health Sciences, University of Edinburgh, Edinburgh, United Kingdom; 3 Division of Epidemiology, Public Health and Primary Care, Imperial College London, London, United Kingdom; 4 National Heart & Lung Institute, Imperial College London, London, United Kingdom; 5 Medical Research Council (MRC)-Asthma UK Centre for Allergic Mechanisms in Asthma, Barts and The London School of Medicine and Dentistry, London, United Kingdom; Medical Research Council, South Africa

## Abstract

**Background:**

There is international interest in enhancing recruitment of minority ethnic people into research, particularly in disease areas with substantial ethnic inequalities. A recent systematic review and meta-analysis found that UK South Asians are at three times increased risk of hospitalisation for asthma when compared to white Europeans. US asthma trials are far more likely to report enrolling minority ethnic people into studies than those conducted in Europe. We investigated approaches to bolster recruitment of South Asians into UK asthma studies through qualitative research with US and UK researchers, and UK community leaders.

**Methods and Findings:**

Interviews were conducted with 36 researchers (19 UK and 17 US) from diverse disciplinary backgrounds and ten community leaders from a range of ethnic, religious, and linguistic backgrounds, followed by self-completion questionnaires. Interviews were digitally recorded, translated where necessary, and transcribed. The Framework approach was used for analysis. Barriers to ethnic minority participation revolved around five key themes: (i) researchers' own attitudes, which ranged from empathy to antipathy to (in a minority of cases) misgivings about the scientific importance of the question under study; (ii) stereotypes and prejudices about the difficulties in engaging with minority ethnic populations; (iii) the logistical challenges posed by language, cultural differences, and research costs set against the need to demonstrate value for money; (iv) the unique contexts of the two countries; and (v) poorly developed understanding amongst some minority ethnic leaders of what research entails and aims to achieve. US researchers were considerably more positive than their UK counterparts about the importance and logistics of including ethnic minorities, which appeared to a large extent to reflect the longer-term impact of the National Institutes of Health's requirement to include minority ethnic people.

**Conclusions:**

Most researchers and community leaders view the broadening of participation in research as important and are reasonably optimistic about the feasibility of recruiting South Asians into asthma studies provided that the barriers can be overcome. Suggested strategies for improving recruitment in the UK included a considerably improved support structure to provide academics with essential contextual information (e.g., languages of particular importance and contact with local gatekeepers), and the need to ensure that care is taken to engage with the minority ethnic communities in ways that are both culturally appropriate and sustainable; ensuring reciprocal benefits was seen as one key way of avoiding gatekeeper fatigue. Although voluntary measures to encourage researchers may have some impact, greater impact might be achieved if UK funding bodies followed the lead of the US National Institutes of Health requiring recruitment of ethnic minorities. Such a move is, however, likely in the short- to medium-term, to prove unpopular with many UK academics because of the added “hassle” factor in engaging with more diverse populations than many have hitherto been accustomed to.

*Please see later in the article for the Editors' Summary*

## Abstract 2

Aziz Sheikh and colleagues report on a qualitative study in the US and the UK to investigate ways to bolster recruitment of South Asians into asthma studies, including making inclusion of diverse populations mandatory.

## Editors' Summary

**Background:**

In an ideal world, everyone would have the same access to health care and the same health outcomes (responses to health interventions). However, health inequalities—gaps in health care and in health between different parts of the population—exist in many countries. In particular, people belonging to ethnic minorities in the UK, the US, and elsewhere have poorer health outcomes for several conditions than people belonging to the ethnic majority (ethnicity is defined by social characteristics such as cultural tradition or national origin). For example, in the UK, people whose ancestors came from the Indian subcontinent (also known as South Asians and comprising in the main of people of Indian, Pakistani, and Bangladeshi origin) are three times as likely to be admitted to hospital for asthma as white Europeans. The reasons underpinning ethnic health inequalities are complex. Some inequalities may reflect intrinsic differences between groups of people—some ethnic minorities may inherit genes that alter their susceptibility to a specific disease. Other ethnic health inequalities may arise because of differences in socioeconomic status or because different cultural traditions affect the uptake of health care services.

**Why Was This Study Done?:**

Minority ethnic groups are often under-represented in health research, which could limit the generalizability of research findings. That is, an asthma treatment that works well in a trial where all the participants are white Europeans might not be suitable for South Asians. Clinicians might nevertheless use the treatment in all their patients irrespective of their ethnicity and thus inadvertently increase ethnic health inequality. So, how can ethnic minorities be encouraged to enroll into research studies? In this qualitative study, the investigators try to answer this question by talking to US and UK asthma researchers and UK community leaders about how they feel about enrolling ethnic minorities into research studies. The investigators chose to compare the feelings of US and UK asthma researchers because minority ethnic people are more likely to enroll into US asthma studies than into UK studies, possibly because the US National Institute of Health's (NIH) Revitalization Act 1993 mandates that all NIH-funded clinical research must include people from ethnic minority groups; there is no similar mandatory policy in the UK.

**What Did the Researchers Do and Find?:**

The investigators interviewed 16 UK and 17 US asthma researchers and three UK social researchers with experience of working with ethnic minorities. They also interviewed ten community leaders from diverse ethnic, religious and linguistic backgrounds. They then analyzed the interviews using the “Framework” approach, an analytical method in which qualitative data are classified and organized according to key themes and then interpreted. By comparing the data from the UK and US researchers, the investigators identified several barriers to ethnic minority participation in health research including: the attitudes of researchers towards the scientific importance of recruiting ethnic minority people into health research studies; prejudices about the difficulties of including ethnic minorities in health research; and the logistical challenges posed by language and cultural differences. In general, the US researchers were more positive than their UK counterparts about the importance and logistics of including ethnic minorities in health research. Finally, the investigators found that some community leaders had a poor understanding of what research entails and about its aims.

**What Do These Findings Mean?:**

These findings reveal a large gap between US and UK researchers in terms of policy, attitudes, practices, and experiences in relation to including ethnic minorities in asthma research. However, they also suggest that most UK researchers and community leaders believe that it is both important and feasible to increase the participation of South Asians in asthma studies. Although some of these findings may have been affected by the study participants sometimes feeling obliged to give “politically correct” answers, these findings are likely to be generalizable to other diseases and to other parts of Europe. Given their findings, the researchers warn that a voluntary code of practice that encourages the recruitment of ethnic minority people into health research studies is unlikely to be successful. Instead, they suggest, the best way to increase the representation of ethnic minority people in health research in the UK might be to follow the US lead and introduce a policy that requires their inclusion in such research.

**Additional Information:**

Please access these Web sites via the online version of this summary at http://dx.doi.org/10.1371/journal.pmed.1000148.

- Families USA, a US nonprofit organization that campaigns for high-quality, affordable health care for all Americans, has information about many aspects of minority health in the US, including an interactive game about minority health issues
- The US Agency for Healthcare Research and Quality has a section on minority health
- The UK Department of Health provides information on health inequalities and a recent report on the experiences of patients in Black and minority ethnic groups
- The UK Parliamentary Office of Science and Technology also has a short article on ethnicity and health
- Information on the NIH Revitalization Act 1993 is available
- NHS Evidences Ethnicity and Health has a variety of policy, clinical, and research resources on ethnicity and health

## Introduction

There is now a considerable body of evidence to show that minority ethnic people in the UK, US, and many other countries have overall poorer health outcomes for a range of conditions than the majority population [1]. Although the reasons underpinning these inequalities are complex and multifaceted, one approach that could be important in helping redress these inequalities is that of focusing research attention on these high risk populations. Developing strategies to involve minority ethnic people in research is hence increasingly being seen as a challenge for multi-ethnic societies; this need is most acutely being recognised in disease areas in which there are known ethnic inequalities in health outcomes [2]. Asthma is one such disease area, as work has shown that UK people of South Asian (where South Asians are defined as people whose ancestry is in the countries of the Indian subcontinent, including India, Pakistan, Bangladesh, and Sri Lanka) and Black:African Caribbean origin are, compared with white Europeans, at significantly increased risk of admission for asthma (South Asians, odds ratio [OR] 2.9, 95% confidence interval [CI] 2.4–3.4 and Black: African Caribbean, OR 2.1, 95% CI 1.8–2.5) [3].

Evidence from the UK, however, indicates that minority ethnic people are markedly under-represented in research, raising important ethical and legal concerns and also potentially limiting the generalisability of study findings [4]–[9]. Comparative US-European data reveal that minority ethnic people are significantly more likely to be recruited into asthma trials in the US than in Europe (62.9% versus 2.9%; *p*<0.0001) [10].

It has been suggested that these differences may be due to differences in research policy between the US and UK, in particular the possible impact of the National Institutes of Health's (NIH) 1993 Revitalization Act (see Box 1) [11]. Equally there may be other important factors such as differing migration contexts and histories, demographic profiles, and broader linguistic and religious considerations. For example, non-white ethnic minorities account for over 30% of the US population [12] compared to an overall proportion of approximately 8% of the UK population (although there are cities such as London and Birmingham where the proportion is much higher) [13]. Not engaging such a large proportion of the population potentially has far greater consequences than a relatively smaller population as is the case in the UK. Also potentially relevant is that the UK has a National Health Service (NHS) which is “free at the point of delivery,” in contrast with the US, which has a private health care system.

**Box 1. Key Features of NIH Policy in Relation to Recruitment of Women and Minorities**

The 1993 NIH Revitalization Act (enforced from 1994 and revised in 2001) resulted in the NIH instituting a policy that “requires all grants, contracts, and intramural projects conducting clinical research to address the Inclusion of Women and Minorities.” The NIH defines clinical research as: “(1) Patient-oriented research. Research conducted with human subjects (or on material of human origin such as tissues, specimens and cognitive phenomena) for which an investigator (or colleague) directly interacts with human subjects. Excluded from this definition are in vitro studies that utilize human tissues that cannot be linked to a living individual. Patient-oriented research includes: (a) mechanisms of human disease, (b) therapeutic interventions, (c) clinical trials, or (d) development of new technologies. (2) Epidemiologic and behavioral studies. (3) Outcomes research and health services research.”

The NIH policy places a responsibility on principal investigators to “assess the theoretical and/or scientific linkages between gender, race/ethnicity and their topic of study” in order to:

- “ensure that women and members of minorities and their subpopulations are included in all human research;
- for Phase III clinical trials, ensure that women and minorities and their subpopulations must be included such that valid analyses of differences in intervention effect can be accomplished;
- not allow cost as an acceptable reason for excluding these groups; and
- initiate programs and support for outreach efforts to recruit these groups into clinical studies.”

We sought to understand possible reasons explaining these differences in recruitment rates between the UK and US and to offer insights to help guide the development of strategies to facilitate the recruitment of minority ethnic people into future UK studies by undertaking a qualitative case study focusing on the UK's South Asian population in the context of asthma research. This group was selected because South Asians now represent the UK's largest minority ethnic grouping and it is the population for which inequalities in asthma are best described and most pronounced.

## Methodology and Methods

### Ethics Statement

Ethics approval was obtained from St Mary's Hospital research ethics committee and research governance approvals were obtained from London, Brent, Harrow, Lothian, Tower Hamlets, Barts and The London and Charing Cross and Westminster research and development boards. Signed informed consent was obtained from all participants.

### Methodology

In order to explore these considerations we decided on using a qualitative interpretivist approach, as we were particularly interested in identifying and understanding people's ideas, experiences, and perceptions on the importance (or otherwise) of this subject [14],[15]. Our investigations consisted of exploring the views of UK-based asthma researchers from a range of relevant disciplinary backgrounds, and the experiences of US researchers as a comparator, because of the potential comparison in highlighting the impact of the different demographic, political, socioeconomic, and legal contexts between these two countries. In addition, we conducted interviews with UK community leaders and focus groups with South Asian people with asthma to understand their experiences and views surrounding this issue. This article, which focuses on the policy relevant data, draws on interviews with academics and community leaders (the data from the focus groups with people with asthma will be reported separately and will not be considered further in this paper).

### Study Design

In-depth interviews were conducted with asthma researchers from the UK and US. We provided a supplementary questionnaire to researchers and the invitation to post anonymised comments onto a Web site, thereby providing the opportunity to offer additional comments confidentially. In addition, we interviewed UK-based South Asian community leaders. Table 1 summarises the techniques used to generate data from these groups of participants.

**Table 1:** Participants and methods for data generation.

Participants	Data Generation Technique	Numbers Approached	Numbers in Final Dataset
Asthma researchers	Interview	43	36 interviews
	Supplementary questionnaire		26 supplementary questionnaires
Community leaders	Interview	11	10

### Sampling and Recruitment Procedures

#### US and UK researchers

A database of principal investigators of recent (2001–2006) asthma projects was compiled through conducting Medline searches, searching the databases of the US NIH and UK National Research Register, Asthma UK, and the Cochrane Airways Group, and contacts with experts. Purposeful sampling was employed to identify researchers from a wide range of disciplinary backgrounds (i.e., genetics, basic sciences, epidemiology, statistics, primary and secondary care, and quantitative and qualitative research), and likely expertise in and experiences of recruiting minority ethnic people into research. We constructed a sampling matrix and began by recruiting broadly across these potentially relevant data fields and then sampling to fill in any important gaps; subsequent sampling was then guided by the emerging findings.

#### UK community leaders

We purposefully recruited South Asian “community leaders” ensuring that we had males and females, those from key relevant ethnic (i.e., Indian, Pakistani, and Bangladeshi) and faith (i.e., Hindu, Muslim, and Sikh) backgrounds, and those occupying formal and informal national and more local UK leadership roles. Ethnic and faith identity were self-described by participants. Study information materials were translated into the main relevant languages (i.e., Hindi, Urdu, Gurmukhi, Gujarati, and Bengali).

### Data Generation and Analysis

Interviews with researchers and community leaders were conducted either face-to-face or by telephone by an experienced qualitative researcher and social anthropologist who is of Indian Muslim Gujarati origin (LH). Topic guides, which were developed through our readings of the academic and policy literature and previous experiences of undertaking research with minority ethnic populations over a number of years, were used to help guide discussions (see Texts S1 and S2); care was taken, however, to ensure that these did not inappropriately constrain discussions. Interviews lasted 15–60 min. Interviewees were given the option of receiving a copy of the transcript. Because of a concern that the researcher participants might be reluctant to disclose their true feelings on a potentially sensitive subject, they were also given the opportunity to complete a confidential questionnaire after the interview, with the added option of posting anonymised comments onto a closed Web site (see Text S3).

Face-to-face and telephone interviews were digitally recorded, translated where necessary, and transcribed together with accompanying field notes. Data were analysed using the Framework approach, a method developed for social policy research and particularly suited to handling large datasets [16]. The following key stages to analysis were included: familiarisation; identifying a thematic framework; indexing; charting; mapping; and interpretation. Several techniques were used to ensure procedural clarity and systematic, verifiable, approaches to analysis; these included consistent availability of topic guides, digital audio-recording, independent preparation of the verbatim transcripts, checking of the translations against the original audio-file, standardised coding and analysis of the data, and the creation of an analysis audit trail to document analytic decisions. The process of data analysis was iterative, in which issues raised by participants (e.g., “critical incidents”) were fed into subsequent interviews; further assisted through weekly discussions between the researcher (LH) and principal investigator (AS), and additional regular discussions of findings with the broader multidisciplinary, multi-ethnic, and multifaith research team with relevant expertise in respiratory medicine, primary care, ethnicity, anthropology, public health, and epidemiology/statistics. Interpretation of findings was enhanced by regular reference to the relevant theoretical and empirical literature on ethnicity and healthcare [4],[5],[8],[17]–[20]. In order to assess the robustness of our findings we actively sought out data offering alternative possible interpretations. Data collection continued to saturation, i.e., the point at which no major new ideas/perspectives were emerging. We anticipated that this would occur after interviews with approximately 30 to 40 researchers and ten to 15 community representatives on the basis of their anticipated more limited experiences of participating in research.

### Reflexivity

Care was taken throughout the process of designing the study, identifying co-investigators, developing data collection techniques, sampling, data generation and analysis to ensure that we considered our own potential biases and that we did not force our own preconceived notions on participants or allow our views unduly to colour our interpretation of these data [21],[22].

## Results

A total of 43 (21 UK and 22 US) invitations were sent out to asthma researchers. Four (two US and two UK) invitees did not respond. Of the 39 that responded, three (US) declined. Given that relatively few UK researchers had any research experience of working with minority ethnic people, we additionally sampled three UK social science researchers with substantive experience of working with minority ethnic people. Thirty-three asthma researchers (16 UK and 17 US) and a further three UK social scientists participated in the study, and of these 26 completed the supplementary questionnaire. Researchers were recruited from a wide range of disciplinary backgrounds (see Table 2). A total of 11 invitations were sent out to community leaders, of whom one declined. Ten interviews were conducted with community leaders; our sample included males and females from diverse ethnic and religious backgrounds (see Table 3).

**Table 2:** Characteristics of asthma researchers.

Researcher Number	Location	Recruited/Targeted Ethnic Minorities	Discipline
AR01	UK	No	Primary care
AR02	US	Yes	Respiratory consultant
AR03	UK	Yes	Social scientist
AR04	UK	No	Primary care
AR05	UK	No	Environmental and occupational medicine
AR06	UK	No	Primary care
AR07	UK	No	Basic scientist/immunologist
AR08	UK	Yes	Respiratory consultant/journal editor
AR09	US	Yes	Allergy and immunology physician
AR11	UK	Yes	Primary care
AR12	US	Yes	Primary care
AR13	UK	No	Primary care
AR14	UK	Yes	Primary care
AR15	UK	No	Epidemiologist
AR16	US	Yes	Respiratory physician/journal editor
AR17	UK	Yes	Health policy and health education
AR18	UK	Yes	Social scientist
AR19	US	Yes	Epidemiologist
AR20	UK	No	Epidemiologist/basic scientist/geneticist
AR21	UK	Yes	Social scientist
AR22	UK	Yes	Basic scientist
AR23	UK	Yes	Epidemiologist
AR24	UK	No	Geneticist
AR27	UK	Yes	Basic scientist/epidemiologist
AR29	US	Yes	Statistician
AR30	US	Yes	Epidemiologist/geneticist
AR35	US	Yes	Epidemiologist/health educationist
AR37	US	Yes	Sociologist/behavioural scientist
AR38	US	Yes	Epidemiologist/statistician
AR39	US	Yes	Translational scientist
AR40	US	Yes	Clinical professor
AR41	US	Yes	Basic scientist
AR42	US	Yes	Pharmacist/health economist/editor
AR43	US	Yes	Health educationist/qualitative researcher
AR44	US	Yes	Basic scientist
AR45	US	Yes	Psychologist/educationalist

Apart from the three UK social scientists, all the interviewees have worked and published on asthma. Most of them have interests in overlapping areas of health research. The three social scientists were chosen because of their interest and work on ethnicity and health research. Identifiers have been kept to a minimum to avoid the risk of inadvertent disclosure of identity.

**Table 3:** Characteristics of community leaders.

Community Leader Number	Ethnic/Religious Background	Leadership Role
CL02	Pakistani Muslim	Local role; formal and informal positions
CL03	Indian Muslim	National role; volunteer
CL04	Indian Muslim	Local role; volunteer; no formal position
CL05	Bangladeshi Muslim	National role; volunteer
CL06	Indian Hindu	Local role; formal position
CL07	Indian Hindu	Local role; no formal position
CL08	Indian Sikh	National and local roles; volunteer
CL09	Indian Muslim	Local role; volunteer
CL11	Indian Hindu	National role; formal position
CL13	Indian Sikh	Local role; formal position

Identifiers have been kept to a minimum to avoid the risk of inadvertent disclosure of identity.

We first discuss the data arising from the perspectives of asthma researchers and then proceed to consider the findings from the community leader interviews.

### Asthma Researchers

#### Interview data

Key issues to emerge from these interviews that can help to explain the differences in recruitment rates between the UK and US include: the importance assigned by researchers to the issue of recruiting minority ethnic people; stereotypes and prejudices; different political contexts; and, above all, the impact of the NIH's policy in the US [11]. We consider each of these subject areas in turn.

#### Attitudes of UK and US researchers towards inclusion and policy considerations

The interviews with UK researchers revealed a wide range of opinions on the subject of minority ethnic recruitment. The views can broadly be divided into three categories: (i) a minority who did not see targeted inclusion of ethnic minorities as having any scientific value except, possibly, in some very specific contexts; (ii) those articulating views that were in general supportive of broader inclusion for most studies (with several exceptions), but with concerns about the practicality of recruiting subjects, viewing the imposition of targets as impractical and even counter-productive; and (iii) those who were committed to a policy of inclusion similar to that introduced by the NIH. The majority of UK researchers tended to fall into the second category, whereas the majority of US researchers tended to fall into the third category.

Some UK researchers displayed a degree of antipathy towards NIH-type targets for inclusion that were perceived to be introduced for political rather than scientific reasons, describing this as “*politicians responding to the political correctness brigade*” (AR05). Another researcher also expressed concern about the NIH targets, commenting “*I think it is probably pandering to political correctness*” (AR23); after a discussion on the pros and cons of mandatory targets, the researcher concluded, “*…so I think I would not be in favour of …funder led guidelines.*” The following parody, introduced in a discussion on the relative merits of positive discrimination, reflects some of the concerns that were expressed: “*So I've got two to go, I need one fat white bald smoker and I need one thin young Asian woman, non-smoker … maybe we should be recruiting more people with multi-pathologies. I bet we don't have enough hypertensives in our asthma studies*” (AR13). This researcher went on to emphasise that the issue was: “*Not just about ethnic minorities!*”. Such views were barriers to more inclusive recruitment practices and one researcher suggested that “*it has to be a societal change and most of these changes cannot come by enforcing it, it comes by people wanting to change it*” (AR39). The practices of a number of UK researchers pointed towards lack of commitment, interest, or will. For most researchers, “*the issue hasn't been given much thought*” (AR24). As one researcher said, “*not recruiting minorities is sort of left over from having a very cohesive mono-culture*” (AR29).

In contrast, US researchers were, on the whole, more positive about the importance of recruiting minority ethnic people on scientific grounds than their UK counterparts as reflected, for example, in the views of this researcher: “*I think there is now a strong likelihood that we will discover important differences and for that reason I think it is important to try to push [for inclusion of minority ethnic people]*” (AR41).

#### Stereotypes and prejudices

Our study also found evidence that some UK researchers' perceptions of, and attitudes towards, ethnic minorities may have played a role in influencing recruitment decisions, some of which are considered below.

Reflecting on some of the barriers to participation in studies or engaging with research, one researcher drew on his own experiences of caring for large numbers of South Asians in his practice; “*First generation migrants direct from the Indian sub-continent tend not to have the skills …to deal with and process information in a digestible form*” (AR14). Earlier on in the interview, this researcher had however pointed to the differences in trying to engage with more established subgroupings within the South Asian population, this in turn reflecting their integration within society as a whole: “Their [Indian] children move fast, because when they came to England. Most of them got jobs, they like the Bengalis they have a big sense of family, but unlike the Bengalis they do their best to ensure that their children will have a good education and go to university and get a good job. Whereas the Bengalis don't really give a s***!”

One researcher, in the context of a discussion contrasting Western European philosophies and society with Eastern self-interested societies, presented ethnic minorities as lacking altruism saying: “*They are more orientated towards their family and less towards society as a whole, or possibly, which is even worse, that they are only willing to contribute to the society where they come from…*” (AR15). He went onto describe South Asian people as “*…a little bit selfish*,” this reflecting, more generally, their perceived relative lack of engagement in society.

There was also a feeling among some researchers that ethnic minorities may not comply with instructions and that they are unreliable, which was raised in the context of a discussion on illegal immigration and the use of pseudo-names: “*When you have people who are unreliable for whatever reason, they should be excluded and that's not a racial thing, it is just a judgement on reliability*” (AR05).

In contrast, the attitude of most US researchers is reflected in the views of these researchers:

“*We have to accept that these people [ethnic minorities] are part of the population and that means respect and it means learning to live with diversity and pluralism*” (AR35).

*“And I think the other thing is, and again this sounds very simple, I think it's just, you know, as you would treat anybody, you treat them with considerable dignity…And I think that becomes very, very important and you recognise their special needs…Very, very important. And you try to be obliging without being overbearing”* (AR27).

The numerous challenges associated with recruiting ethnic minorities led several UK and US researchers, irrespective of their personal commitment to the idea, to view it as a major “*hassle*” with considerable implications in relation to “*time*, *effort and resource*” (AR05). There was, in general, also considerably more optimism from US researchers when compared to UK researchers regarding their ability to engage with minority communities, which seemed to be a reflection of both greater commitment and the confidence resulting from previous successful engagements. Several researchers thus noted that the logistical barriers were not insurmountable, their experiences indicating that ethnic minorities do participate if appropriately approached. As one US researcher noted:

“*We may, we'll make up to 50, 60 telephone calls trying to get somebody. I mean, this is extremely labor intensive. We will, if we get set someone enrolled and we need to collect data and they can't do it by telephone, we send someone to their house. We take it very, very seriously…it's not just a matter of ringing someone up once or twice and if it doesn't work calling it a day. You know, you have to be ready, and this costs money…*” (AR02).

This view of increased costs associated with recruiting minority ethnic people was, although widespread, not universally shared by US researchers. One researcher, for example, who was committed to the importance of inclusiveness in research, when asked about increased costs associated with recruiting minority ethnic people, retorted: “*I don't believe any of that!*” (AR38).

Finally, US researchers frequently expressed concern at the possibility of excluding minority ethnic people, as for example, reflected by this researcher: “*You know, most – I find it hard to… I find it hard to accept there are researchers who will not work with an Asian population or an ethnic diverse population or however you want to group them*” (AR35).

#### Demographic, political, and socioeconomic contexts of the two countries

Both US and UK researchers either pointed out or accepted the fact that the demographic profile of ethnic minorities, their histories, political engagement, influence, and the way the health services are configured in the two countries also contribute to differential recruitment rates in the two countries. For example, one participant said: “*the Tuskegee study is still a legacy that sticks with our community in terms of research particularly when a lot of our researchers are White*” (AR37) [23],[24].

The US population is largely served by the private health sector through insurance. As minorities are less likely to be insured, participating in an asthma study potentially has the added advantage of receiving free medical attention making it more attractive for minorities in the US to participate in research:

“*Most of our people living in the inner city have you know, government supported insurance….So that helps that it means they'll get their drugs covered, you don't charge for visits, you know, that's [part of the study and in some of the studies we actually provide them with medication so they don't actually have to go to the pharmacy and deal with the hassle*”(AR27).

Such incentives in some instances do not motivate interest in participating in studies: “*…a lot of times we have difficulty getting White patients because they don't need the medication. Why would they take two hours out of their day to drop downtown and do this research study?*” (AR37).

#### NIH policy and its impact on attitudes and experiences in the US contrasted with the UK

Our data suggest that the introduction of the NIH policy had a major impact on the attitudes and experiences of US researchers and probably explains much of the differences in perspectives and experiences noted above. Salient features of the NIH policy are summarised in Box 1 [11].

We found that most US asthma researchers currently accept the stipulation of the NIH policy and had as a consequence devised creative strategies to address the challenge of recruiting marginalised groups including, for example, community leaders being “*hired as study personnel*” so that they in effect became study employees, thereby making it *“legitimate to pay them”* and cooperating with other research teams “*so, when a racial or ethnic difference seems to be important, we co-operate with another area that has subjects we can recruit*” (AR29). There was an expressed greater willingness to work with people “*in their own territory,*” including going into “*their own homes or in a community centre*” or hosting free “*community events*” such as “*barbecues*” (AR35) or setting up study clinics in accessible places such as “*in a suburban shopping mall or some place that's right next to the bus stop or tube stop or…they can just walk right in. Or they can just drive right up and walk right in to see you*” (AR38).

There were also instances in which the NIH took a more proactive role:

“*I'm thinking of our first study where we really were working in a school and kind of recruiting what you would call difficult populations. We were lucky because it was a contract from NIH where there were several people that were contracted to do the same type of work and NIH got us together on a regular basis and I think we were able to help each other*” (AR37).

The strategies adopted were typically resource and time intensive as well as diverse as researchers tailored their approaches to take into account both the nature of the study they were conducting and the social context within which they were working.

Though only NIH funded studies are required to recruit and report on ethnicity, the policy appears to have had a wider impact beyond NIH funded studies. “*It's pretty much an expectation so I think it really does filter down if you, … have a similar organization that's well respected I think people start to look at that [NIH policy] as really the gold standard*” (AR37). Thus although pharmaceutical companies are not bound by quotas they are answerable to the Food and Drug Administration (FDA), which requires “…*evidence that the drug's not acting differently in one group than another*” (AR29).

There was resentment in the US when NIH policy was first introduced as it was seen as a political move. “*I think it was entirely political…*” (AR41). “*They [researchers] hated it … and they wrote every excuse in the book of why they shouldn't have to do it*” (AR12). “*It was clearly difficult for us in the beginning*” (AR41). However, with new evidence emerging, there seems to be more conviction in the US. “*It's hard to argue that having a reasonable amount of demographic diversity isn't scientifically revealing*. *I think now there is a strong likelihood that we will discover important differences and for that reason I think it's important to try to push even if it's a political argument for pushing*” (AR41).

A few researchers felt that it would be better if they did not have to pursue targets for certain studies. Nevertheless, not a single US researcher questioned the benefits of minority inclusion or called for it to be eliminated. In spite of the apparent commitment, it was felt by some that “*they'd go right back*” (AR12) if the NIH no longer insisted on the need for recruitment of minority people.

The overall importance and impact of the NIH policy was well summarised in the following words:

*“All of us who are working with NIH grants, we have to indicate the percentage of minority people that will be involved in our grants and we have to report on a quarterly basis how we are doing on recruitment. And if we're not doing well on recruitment we hear from our programme officer and one can lose a grant if recruitment isn't as it needs to be and this is because NIH had a lot of difficulty in years past with giving grants and at the end people would say, well you know what, we just couldn't recruit the people and so NIH said well we don't think it's a good investment of our money…”* (AR02).

Unlike the US, there is no comparable policy existing in the UK. Most UK researchers did not believe that existing laws and guidelines required them to include ethnic minorities in their study though some did express the view that the Race Relations Amendment Act [25] and the Human Rights Act [26] clearly puts the onus on researchers to ensure that their study sample is representative of the population under study and that no group is excluded from the benefits of research.

UK researchers did not specifically target or monitor ethnic minorities unless the study question specifically required them to do so. Most researchers “*just simply advertise*” (AR20) and “*recruit people regardless of their ethnicity as they come through the clinics*”(AR24) and have not “*particularly monitored the ethnic minorities within the group … [they] used*” (AR07). “*As long as you capture the data that's fine, but to deliberately go out and say, ‘I must recruit X number of Asian …’ just doesn't make sense*” (AR05).

The standard response, even from those who whole-heartedly supported the idea of inclusion, was that this consciousness was not translated into action in terms of research strategies. “*We never put positive discrimination … you know we haven't done historically … you get people who come*” (AR17).

#### Questionnaire data

Supplementary questionnaires (see Text S3) were completed by 26 researchers (72% overall: 74% UK and 71% US). The findings were in close agreement with those elicited through interviews but did offer a few additional insights into the barriers facing researchers in recruiting minority populations and the observation that UK researchers perceived that this subject was a relatively unimportant issue for funders and journals. The majority of UK researchers in support of this position and who thought this issue was important for journals and funders were themselves from social science backgrounds and/or from minority ethnic groups, whereas researchers in general in the US considered this to be a particularly important issue for funding bodies. None of the respondents expressed a wish to post anonymised comments onto a Web site.

### Perspectives of UK Community Leaders

The main themes to emerge from the interviews with community leaders included a lack of awareness of and/or opportunities to participate in research, a general expressed willingness to get involved if invited to do so, particularly if cultural sensitivities and logistical considerations were adequately attended to. There was, however, some concern from those who had helped facilitate research that involvement carried opportunity costs, which were not always adequately recognised or reciprocated. These issues have policy implications in relation to the skills and resources researchers need to make such relationships mutually fulfilling.

#### Lack of awareness of research and opportunities to participate

A number of the community leaders had little experience of being asked to help recruit participants or, for that matter, personally being invited to participate in research, whether in the context of asthma or indeed any other research.

“*Well first of all I think the most important thing is the media. It should be put through the media because people must know what is asthma. The Asian community and where they can go and how they can come forward if there's any research going on. Nobody knows about it. I don't know anything about it. Personally I don't know anything about it unless somebody approached me and talked to me to tell me what it is, then I would know it*” (CL03).

This lack of engagement with research appeared to be widespread, often resulting in the lack of any broader opportunities to learn informally through friends and family members, for example about research participation and what it might entail.

*I: Have you ever been approached for any health research projects?*

*CL02: No.*

*I: Never?*

*CL02: No.*

*I: …Do you know of anyone who's ever participated in health research?*

*CL02: No, not that I know of, no.* (CL02)

Those who had some connection with the medical profession, either themselves being doctors or working with medical colleagues or through their respective organisations, were in contrast more likely to have been approached to help recruit participants, although such approaches tended to be relatively infrequent. Given the relatively high proportion of South Asian doctors in the UK, drawing on the support of such individuals could prove very useful, although there is the associated risk of possible gatekeeper fatigue (discussed below).

#### Recognition of the importance of this field and a willingness to engage if invited to do so

There was widely held and strongly expressed support for the involvement of minority ethnic communities in research, which was argued for on the grounds of fairness and justice, the need to reduce the high and disproportionate levels of morbidity, and, more generally, in order to better understand the changing nature of minority ethnic communities. Responding to a question on the appropriateness or otherwise of this enquiry, one leader, for example, commented that such studies were: “*Oh absolutely essential. I think one of the problems is that we don't know enough formalised studies. We don't know enough about people generally, you know, what they think, how they're perceived and so forth…*” (CL04).

There was amongst some leaders, although certainly not all, a detailed appreciation of discrimination and equality legislation, which they were able to draw on to inform their responses in the context of discussions on research. Reference was in this respect made to, amongst other considerations, “*the Race Equality Scheme*” and the “*Commission for Equality and Human Rights*” (CL11), which were mentioned to underscore the importance of fairness for all members of society [25],[26].

A sense of responsibility towards community members also served as an important motivating factor:

“*But there is a lot of ways we can help you. We will do our best to assist you in whatever way we can. We are here to see our community benefit. Doesn't matter whether it's Pakistani, Indian, Bengali. We work with every one of them. So we will try and help as much as possible from this office. So our doors are open to you at any time*” (CL03).

Perhaps, ever more telling, was that a number of the community leaders interviewed went out of their way to help recruit people with asthma and carers for the focus group component of this work.

#### Recognising the need to ensure cultural sensitivity and opportunity costs

There were a number of suggestions made on how recruitment might best be encouraged centring on the need to ensure that the language needs of minority ethnic communities were adequately met and that cultural and religious and cultural values, such as the need for gender segregation, were respected:

“*Particularly, if you're handling the women, you have to be very careful. You know that, in Islam, there are certain things that the women don't like. They'd rather they be handled by a female doctor, rather than handled by a male doctor*” (CL03).

The need to think about convenience, recognising that people had busy lives, was also emphasised particularly as many are disadvantaged and hence would find it difficult to meet transportation costs, for example. Interviewees also reflected on the lack of capacity that their respective organisations had to facilitate such additional noncore work and that if they did engage with such requests this would have opportunity costs.

“*Many mosques and centres don't have that capacity because of the constraint in financial sources…they don't have enough people…we are encouraging our mosques and centres to work with the local community, local council, local service providers like hospitals and others and it's gradually getting through, but there has to be reciprocal attempt from the service provider*” (CL05).

Finding “*a carrot*” of some sort for the organisation was suggested as potentially important in helping to address such barriers; the community organisations' needs in this respect tended to be relatively modest such as “*hall hire…for a Sunday afternoon*” or paying for “*a dinner*” (CL06). Although these suggestions were in keeping with the types of activities already being undertaken by many US researchers, such initiatives would require new ways of working by UK academics and funding bodies, as such activities have funding implications that are typically not budgeted for in research applications.

One of the leaders who had the most experience of facilitating research in the past was, while retaining appreciation of the importance of the subject, very negative about many of her own organisation's experiences as the approaches were often seen as “*tokenistic*” or “*last gasp*” attempts when other approaches had failed. This then led her to question her personal involvement: “*You know after years and years of taking part and then thinking ‘Well you know what does happen with all that stuff?’ Nobody ever gets back to us about it…So since then I have been very cynical and very careful and I ask a lot of questions and I would want to know what it is that we are going to get out of this*” (CL13).

## Discussion

This study has revealed a wide gap between the US and UK in terms of policy, attitudes, practices, and experiences in relation to the inclusion of minority ethnic people in asthma research. These differences should not, however, mask the broader convergence of policy and scientific interest in relation to the question of inclusiveness of diverse populations, which was evidenced in the views expressed by a number of US and UK academics. There was also a similarity in views on the logistical and resource implications of broadening recruitment beyond the majority white population. Whereas the NIH policy appears to be a major driving force for the more inclusive ethos in the US, the absence of such a policy in the UK coupled with antipathy, inexperience, and apprehension contribute towards the marked relative under-representation of ethnic minorities in UK asthma studies. Our findings suggest that a US-style legislative-based approach could, if suitably adapted for a UK context, bolster recruitment of minority ethnic people into UK research, both in relation to asthma and possibly in other areas. Focusing attention on this issue would also, it seems, eventually promote engagement of researchers with minority ethnic communities in mutually respectful and fulfilling ways.

### Strengths and Limitations of This Study

This is the first trans-Atlantic study of its kind, to our knowledge, which builds on previous quantitative work and consequently sheds light on a question that we believe is of international importance. This study complements the previous descriptive work and offers insights into why the now well-described differences in recruitment rates between the US and UK exist. Given the nature of the insights obtained and that these differences between the US and UK have also been noted in research in conditions other than asthma, our findings are likely to be transferable to other disease areas and contexts [6],[27]. Our previous work has shown that UK researchers are probably more aware of this issue of ethnic representation than researchers in many other parts of Europe; therefore we suspect these findings will also be transferable to other parts of Western Europe [5],[6],[10].

There is a small, but nonetheless inevitable risk that, given the sensitive nature of the question under study, participants may have at times felt obliged to give “politically correct” answers. On a related point, the views of the research team on the importance of thinking about ethnicity considerations in the context of research are well known and so these views could also have acted as a bar to frank discussion. We anticipated the potential importance of these issues and so took care to ensure that interviews were conducted in a nonjudgmental manner thereby allowing free and frank conversation; we made clear to participants that members of the research team other than the interviewer would only have access to anonymised material, and offered interviewees the option of completing an electronic questionnaire (together with the option of posting completely anonymised comments onto a dedicated Web site) after the interview. Of related importance, care needs to be taken in interpreting the data from questionnaires as they were obtained from nonrepresentative samples, which limits the ability to generalise from these data. Bearing in mind the main aim of the questionnaire, it was encouraging that there were no additional major issues arising that had not previously been covered in the interviews; the absence of researchers' anonymised comments on an offered Web site may suggest that a public forum was superfluous, but it may alternatively reflect the fact that researchers were unconvinced that comments could not be attributed or that they were too busy to engage further with this study.

The difficulties of defining who is and who is not a “community leader” are well recognised. Part of the problem in this respect is the concern that there are sometimes self-defined leaders who have relatively little direct influence on their communities. In order to try and work around this issue we sought to recruit individuals with both formal and informal positions at national and local levels. We were also keen to recruit community leaders from across the three main faith groups of interest—Muslims, Hindus and Sikhs—and for this reason we, in particular, sampled those of Indian background (as the overwhelming majority of Pakistani and Bangladeshi community leaders are Muslims). The interviews with the community leaders in which they expressed a willingness to participate in research need also to be interpreted cautiously as it is well recognised that there is often a gulf between intentions and actual practice; nevertheless, the overwhelming majority of the community leaders who were invited to participate in this study agreed to do so, which bodes well for other similar approaches. Furthermore, many of these individuals actively helped with recruiting people with asthma for the focus groups.

More fundamentally, some may question the main premise underpinning this research, namely that taking ethnicity into consideration when recruiting into research is in itself of importance. It has for example recently been argued by Epstein, among others, that the scientific arguments underpinning this drive to promoting inclusion are of questionable scientific value [28]. Our view, formulated over the course of several years of work in this area, is that contextual considerations are potentially of considerable importance when reflecting on the scientific importance of ethnicity as a variable; overall, however, there is as yet inadequate data to be able to decide which contexts are of greatest importance, and so in order to progress understanding in this field at this stage there is a need to promote greater inclusiveness in research. We also believe that there are important societal gains to be had from promoting inclusion in research in multi-ethnic societies.

We may also be criticised for focusing on South Asians and thereby excluding other minority ethnic populations. Our decision to focus on this population was taken, as discussed in the introduction, on the basis of demographic considerations and also on the well-recognised and persistent asthma inequalities experienced by South Asians. There were, however, also more pragmatic considerations; in particular, the considerable difficulties in obtaining support for research of this kind and in the face of limited resources, the need to begin this research somewhere, while also ensuring that it was undertaken in a way that was sensitive (e.g., in relation to meeting language needs) to the communities under study. Our hope is that in due course we will be able to extend this enquiry to other minority ethnic populations. The focus of this work, which was in relation to bolstering recruitment of minority ethnic people into UK research and the logistical constraints discussed above, also guided our decision to only recruit UK community leaders. Future research could usefully explore the perspectives and experiences of US community leaders.

There is also the risk that through focusing attention on ethnicity that we may inadvertently be exacerbating the problems of marginalisation of these minority communities. Although we acknowledge this as a potential risk, particularly in the short-term, overall we believe that in the medium- to longer-term highlighting exclusion issues will result in more benefit than harm.

Finally, there are, as with all qualitative work, questions about how generalisable the findings are beyond the participants included in this study. The underlying factors identified in explaining these differences do suggest, however, that our findings are potentially transferable to other minority ethnic populations and other disease contexts.

### How These Findings Relate to the Broader Literature

A key question that arose from the literature and our early interviews was whether the NIH guidelines played a central role in the US. It is important in this respect to note that the NIH is the world's biggest research funder with an annual budget of >US$28 billion. Our subsequent data showed that the policy not only played an important role in the way NIH funded research is conducted, but it also appears to have had a ripple effect in relation to non-NIH funded research. This policy seems to have consequently increased researchers' experience, expertise, and confidence in approaching and interacting with “hard to reach” (or alternatively “easy to ignore”) populations, and funders' appreciation of the cost implications of broadening participation. This work echoes the findings of Wendler et al. [29], which demonstrated that the failure to invite participants is an important barrier to participation.

Our data indicate that the possible benefits of participating in research (such as free medical attention and routine use of financial incentives) in the US may make participation attractive to those who are uninsured (more commonly minorities). As there are no such tangible benefits in the UK (the NHS is free and financial incentives are seldom given), participants may not see the same direct benefits. On a related note, there appears to be a general unease about the offer of financial incentives or funding to community groups to host events, which could facilitate recruitment, because of concerns that this may result in coercion to participate. Exclusion of ethnic minorities is, however, contrary to the spirit and letter of the Race Relations (Amendment) Act and Human Rights Acts [25],[26], which highlight the importance of equality of opportunity and respect for individual's beliefs and practices; the NHS Patient's Charter more explicitly gives patients “*…the right to choose whether or not you want to take part in medical research*” [30], something which is currently being withheld from many UK patients. This is also contrary to the UK Department of Health's Research Governance Framework, which also highlights the importance of inclusivity in research [31].

Our findings also resonate with the findings reported by Hussain-Gambles et al. [32],[33], who demonstrated that researchers who do not see the benefits of an inclusive sample and who operate from preconceived notions about a group are unlikely to seek them out as study subjects. We were struck by the extent of stereotyping expressed by some UK researchers, which suggests that these views may not prove easy to challenge or modify in the short-term. More generally, the lack of availability of appropriate diversity training and the limitations of the training options that are available does not help in this respect [34]. Engagement with people from the communities in question—which our data suggest can be achieved though not without incurring costs—will however force the challenging of such stereotypes, and we hope this will eventually result in more nuanced perspectives on these issues. We also hope that over time the increasing move to working in larger research teams will allow researchers to work with colleagues who may have a different set of experiences in this respect. Although some of these comments expressing stereotypical views may be the result of specific experiences, they are most unlikely to be generalisable across an entire ethnic or religious group. Moreover, a community's own experience of marginalisation, and disenfranchisement, even if imagined in some cases, cannot be ignored. More encouragingly, we did uncover at least some examples in the UK where substantial progress has been achieved in reaching out to and engaging with minority ethnic communities and through so doing facilitating their inclusion with research.

### Conclusions and Recommendations

The crucial question from a policy perspective is whether the UK needs or is indeed ready for an NIH-type policy on recruitment of minority ethnic groups. This work demonstrates that such a policy would be unpopular in the UK. However, the US example suggests that if introduced appropriately, initial resentment can give way to conviction and a change of attitudes.

The fact that many other US funding bodies and academic institutions now implicitly require inclusion of minority ethnic people—even though the law does not require them to do so—suggests that there is a degree of commitment to the idea of inclusion among the leaders and policy makers in the US scientific community. An NIH-type policy, backed with legislation and funding and other technical support [35] for researchers, instituted by a UK funding body of national standing—such as the Medical Research Council or the National Institute of Health Research—would, we believe, probably have a major impact on the way research is conducted in the UK. The Research Governance Framework provides an important platform on which to build such UK policy [31].

Given the considerable concerns expressed by UK researchers about any move towards a mandatory NIH-type policy, it might be argued that it is best initially to continue with the UK's voluntary codes of best practice exhorting researchers to recruit minority ethnic people into their studies. However, given the degree of scepticism and worries about logistics identified, and the US experiences, we believe these voluntary codes are unlikely to translate into improved outcomes unless there is considerable accompanying support for researchers both in relation to ready access to expertise and also financial and material support to develop long-term relationships with the communities of interest. For inclusionary recruitment to occur, funding bodies will need to both recognise its importance and appreciate the use of funds being used to support the range of community initiatives necessary to implement research.

If, however, such voluntary measures prove unsuccessful—which is certainly possible—we hypothesise that an NIH-type approach is a credible one that should be considered, as it will most probably translate into substantial improvements in recruitment rates. As with any hypothesis, however, it would need to be tested to examine its credibility and also to ensure that such an initiative does not inadvertently result in more harm than good.

Although the focus of this work was on comparing and contrasting experiences between the UK and US, we suspect that the implications of our findings will also apply to many other multi-ethnic societies. Research funders, policy makers, researchers, and the minority ethnic communities themselves in other parts of the world should therefore consider the implications of our work and, if necessary, critically evaluate and reformulate the recruitment procedures currently being supported and employed.

## Supporting Information

Text S1 Topic guide for interviews with researchers.
(0.03 MB DOC)

Text S2 Topic guide for interviews with community leaders.
(0.03 MB DOC)

Text S3 Supplementary questionnaire for researchers.
(0.03 MB DOC)

## Abbreviations

CI: confidence interval
NIH: National Institutes of Health
OR: odds ratio
