# Supplementary material for: Facilitating the Recruitment of Minority Ethnic People into Research: Qualitative Case Study of South Asians and Asthma
Source: PLoS Med. 2009 Oct 13;6(10):e1000148. doi: 10.1371/journal.pmed.1000148 (PMC2752116; doi:10.1371/journal.pmed.1000148)
Supplement: Text S1 — Topic guide for interviews with researchers. (0.03 MB DOC) [file pmed.1000148.s001.doc]

**Text S1: Topic guide for interviews with researchers**

Introductions

- Particular interest or area of asthma research? (basic sciences, epidemiology, translational work, clinical/applied research, etc.)
- Experience of clinical/research work in US? UK? Elsewhere?

Subject under study

- Understanding of rationale for study
- Is the subject important? Why/why not?
- In what contexts might ethnicity considerations be most relevant?

Experiences of recruiting minority ethnic people with asthma into studies

- Ever considered the question of ethnicity of recruits into studies?
- Experiences of attempting to engage with minority ethnic communities, in particular South Asians.
- Are there barriers to recruiting South Asians that you have personally experienced or you are aware that other researchers have experienced? (Prompts: linguistic, cultural, religious, fear of falling foul, lack of interest, research fatigue, gatekeeper fatigue, obtaining informed consent, time, costs, irritated by political correctness)
- Are there strategies that you/others have successfully used in recruiting South Asians? (Prompts: location of study, minority ethnic co-researcher/research fellow, support of national/local community/religious organisations, financial)

Reporting of data

- When might ethnic specific reporting of data be relevant?
- Have you done this with previous studies? Why/why not?
- Are funders/journals interested?

Next steps

- Is more discussion, debate and consensus still needed?
- Response to recent research showing that minority ethnic people are equally likely to participate as Whites if invited to do so
- Is the US model of target setting a useful way forward?
- National initiatives that might help facilitate greater recruitment of South Asians?
- Actions that funding bodies/ethics committees/journals/researchers/minority ethnic groups need to take?
- Anything else?

Demographic characteristics of participant

- Age
- Ethnicity
- Position
- Geographical location
