# Supplementary material for: Facilitating the Recruitment of Minority Ethnic People into Research: Qualitative Case Study of South Asians and Asthma
Source: PLoS Med. 2009 Oct 13;6(10):e1000148. doi: 10.1371/journal.pmed.1000148 (PMC2752116; doi:10.1371/journal.pmed.1000148)
Supplement: Text S2 — Topic guide for interviews with community leaders. (0.03 MB DOC) [file pmed.1000148.s002.doc]

**Text S2: Topic guide for interviews with community leaders**

Introductions

- Organisation (if any)
- Role within it

Subject under study

- Understanding of asthma
- Understanding of rationale the study
- Is the subject important? Why? Why not?
- How much of a priority is this to you or your organisation?
- In what contexts might ethnicity considerations be most relevant in?

Experiences of being involved with the research studies

- Personal experience
- Friends? Relatives?
- Ways in which approached?
- Positive/negative experiences

Barriers/facilitators to research

- What might be the barriers to recruiting South Asians into research studies? (Prompts: Linguistic? Cultural? Religious? Research is not interested? Lack of interest? Research fatigue? Gatekeeper fatigue? Obtaining informed consent? Time? Cost? Tokenism? Racism?)
- What might be facilitators to successfully recruiting South Asians? (Prompts: Location of study? Minority ethnic co-researcher/research fellow? Support of national/local community/religious organisations? Financial?)

Next steps

- Is more discussion, debate and consensus still needed?
- Response to recent research showing that minority ethnic people are equally likely to participate as Whites if invited to do so?
- Is the US model of target setting a useful way forward?
- National initiatives that might help facilitate greater recruitment of South Asians?
- What might practically support you/your organisation in helping recruitment? Would it/these really make a difference?
- Actions that funding bodies/ethics committees/journals/researchers/minority ethnic groups need to take? Anything else?

Demographic characteristics of participant

- Age
- Ethnicity
- Position
- Geographical location
