# Supplementary material for: Facilitating the Recruitment of Minority Ethnic People into Research: Qualitative Case Study of South Asians and Asthma
Source: PLoS Med. 2009 Oct 13;6(10):e1000148. doi: 10.1371/journal.pmed.1000148 (PMC2752116; doi:10.1371/journal.pmed.1000148)
Supplement: Text S3 — Supplementary questionnaire for researchers. (0.03 MB DOC) [file pmed.1000148.s003.doc]

**Text S3: Supplementary questionnaire for researchers**

**South Asians and asthma research**

*We would be grateful if you would complete this short questionnaire. Your data will be kept confidential and your comments will not be attributed to you.*

1. Do you think the discourse/ debate surrounding recruitment of ethnic minorities into asthma research (or any other medical research) has received too much or too little attention?

Too much/about right/too little/don’t know (delete as appropriate)

Please explain your reasons:

1. Do you believe that including ethnic minorities in research adds value to the research?

Yes/no/don't know (delete as appropriate)

Please explain your reasons:

1. In what types of asthma research might ethnicity considerations be most relevant?
2. Would you support compulsory recruitment and reporting of ethnically diverse populations for all research?
3. Do you think funders and/or journals see this issue as important?

Yes/no/don't know (delete as appropriate)

Please expand:

1. Would you be interested in posting anonymous comments on this subject on a dedicated website?
2. Do you have any other comments?

Kindly return the completed questionnaire to Dr Laila Halani by e-mail fax or post. E-mail: [Laila.Halani@ed.ac.uk](mailto:Laila.Halani@ed.ac.uk) Fax: 0131 650 9119 Postal address: The University of Edinburgh, 20 West Richmond St, Edinburgh EH8 9DX, UK.
